# Deep‐learning predicted PET can be subtracted from the true clinical fluorodeoxyglucose PET co‐registered to MRI to identify the epileptogenic zone in focal epilepsy

**DOI:** 10.1002/epi4.12820

**Published:** 2023-08-29

**Authors:** Anthime Flaus, Julien Jung, Karine Ostrowky‐Coste, Sylvain Rheims, Marc Guénot, Sandrine Bouvard, Marc Janier, Siti N. Yaakub, Carole Lartizien, Nicolas Costes, Alexander Hammers

**Affiliations:** ^1^ Department of Nuclear Medicine Hospices Civils de Lyon Lyon France; ^2^ Medical Faculty of Lyon Est University Claude Bernard Lyon 1 Lyon France; ^3^ King's College London & Guy's and St Thomas' PET Centre, School of Biomedical Engineering and Imaging Sciences King's College London London UK; ^4^ Lyon Neuroscience Research Center INSERM U1028/CNRS UMR5292 Lyon France; ^5^ Department of Functional Neurology and Epileptology, Hospices Civils de Lyon, Member of the ERN EpiCARE Lyon 1 University Lyon France; ^6^ Department of Pediatric Clinical Epileptology, Sleep Disorders, and Functional Neurology Hospices Civils de Lyon, Member of the ERN EpiCARE Lyon France; ^7^ Department of Functional Neurosurgery, Hospices Civils de Lyon, Member of the ERN EpiCARE Lyon 1 University Lyon France; ^8^ Brain Research & Imaging Centre University of Plymouth Plymouth UK; ^9^ INSA‐Lyon, CNRS, Inserm, CREATIS UMR 5220, U1294 University Claude Bernard Lyon 1 Lyon France; ^10^ CERMEP‐Life Imaging Lyon France

**Keywords:** anomaly detection, artificial intelligence, computer‐aided diagnosis, epilepsy, generative adversarial network, nuclear medicine

## Abstract

**Objective:**

Normal interictal [^18^F]FDG‐PET can be predicted from the corresponding T1w MRI with Generative Adversarial Networks (GANs). A technique we call SIPCOM (Subtraction Interictal PET Co‐registered to MRI) can then be used to compare epilepsy patients' predicted and clinical PET. We assessed the ability of SIPCOM to identify the Resection Zone (RZ) in patients with drug‐resistant epilepsy (DRE) with reference to visual and statistical parametric mapping (SPM) analysis.

**Methods:**

Patients with complete presurgical work‐up and subsequent SEEG and cortectomy were included. RZ localisation, the reference region, was assigned to one of eighteen anatomical brain regions. SIPCOM was implemented using healthy controls to train a GAN. To compare, the clinical PET coregistered to MRI was visually assessed by two trained readers, and a standard SPM analysis was performed.

**Results:**

Twenty patients aged 17‐50 (32 ± 7.8) years were included, 14 (70%) with temporal lobe epilepsy (TLE). Eight (40%) were MRI‐negative. After surgery, 14 patients (70%) had a good outcome (Engel I‐II). RZ localisation rate was 60% with SIPCOM vs 35% using SPM (*P* = 0.015) and vs 85% using visual analysis (*P* = 0.54). Results were similar for Engel I‐II patients, the RZ localisation rate was 64% with SIPCOM vs 36% with SPM. With SIPCOM localisation was correct in 67% in MRI‐positive vs 50% in MRI‐negative patients, and 64% in TLE vs 43% in extra‐TLE. The average number of false‐positive clusters was 2.2 ± 1.3 using SIPCOM vs 2.3 ± 3.1 using SPM. All RZs localized with SPM were correctly localized with SIPCOM. In one case, PET and MRI were visually reported as negative, but both SIPCOM and SPM localized the RZ.

**Significance:**

SIPCOM performed better than the reference computer‐assisted method (SPM) for RZ detection in a group of operated DRE patients. SIPCOM's impact on epilepsy management needs to be prospectively validated.

**Key points**

- AI can predict PET from T1w magnetic resonance imaging [MRI] with good accuracy.
- Subtraction of the clinical from the predicted PET (SIPCOM) in analogy to SISCOM.
- SIPCOM performed better than SPM to localize the resection zone in [^18^F]FDG‐PET.
- SIPCOM performance advantages were relatively similar in each sub‐group (MRI positive/negative patients, temporal lobe epilepsy [TLE] vs extra‐TLE patients).
- Visual analysis performed better than SIPCOM except in one case.

## 1 INTRODUCTION

In drug‐resistant epilepsy (DRE), surgery is the only potentially curative treatment.^1^ A prerequisite is the precise identification of the epileptogenic zone (EZ) during a multi‐step presurgical work‐up,^2^ with magnetic resonance imaging (MRI) as the mainstay of imaging. ^18^F‐labeled fluorodeoxyglucose positron emission tomography‐computed tomography ([^18^F]FDG PET‐CT) is a widely used functional imaging modality, which in combination with structural MRI allows correct EZ localisation in approximately two‐thirds of patients^3^, ^4^ and is particularly useful if MRI is noncontributory (negative).^3^, ^5^

Visual assessment remains the preferred method in clinical routine to review [^18^F]FDG‐PET images. However, EZ detection rate is highly variable between studies,^4^, ^6^, ^7^, ^8^, ^9^, ^10^, ^11^, ^12^, ^13^ ranging from 33%^11^ to 95%,^6^, ^14^ with higher detection rates for temporal lobe epilepsy (TLE) vs extra‐temporal lobe epilepsy (ETLE),^9^, ^13^ when lesions can be seen on MRI vs MRI‐negative epilepsy,^4^, ^10^, ^12^, ^15^ and when [^18^F]FDG‐PET is inspected combined with co‐registered MRI vs reading [^18^F]FDG‐PET alone.^7^, ^8^, ^10^, ^13^ Visual assessment performance also varies with reader experience which could lead to significant diagnostic bias,^16^ in addition to difficulty providing consistent advice and scaling up services when needed. Therefore, computer‐aided diagnosis (CAD) methods were developed to facilitate EZ location and improve reliability and reproducibility. Statistical parametric mapping (SPM)^17^ analysis of PET data is the most widely studied^3^ and consists of a voxel‐by‐voxel mass univariate analysis comparison of brain [^18^F]FDG uptake of a patient compared to a group of controls. Its performance for EZ localisation varies widely across studies,^5^, ^6^, ^15^, ^18^, ^19^, ^20^, ^21^, ^22^, ^23^, ^24^, ^25^, ^26^ from 20%^26^ to 86%.^6^ In terms of added value, in one study specifically looking at PET designated as normal on visual analysis by two readers but without coregistration with MRI, SPM was able to locate the EZ determined during presurgical workup in 40%.^22^ Two studies showed a similar trend^5^, ^23^ but several studies did not: in surgically treated adults, SPM tended to be less sensitive than visual analysis for lateralization of TLE^18^ and for localization of ETLE.^15^, ^19^ In children, for surgically treated TLE, visual analysis tended to outperform SPM to localize EZ.^6^ In a mixed cohort of surgically treated TLE and ETLE, visual analysis and SPM were complementary, as SPM performed better for medially located EZs and visual analysis performed better for lateral hypometabolism.^20^

Recently, deep learning (DL) based methods for abnormality detection on [^18^F]FDG PET have emerged. They are mainly based on auto‐encoders, using latent space representation of the FDG normal distribution to detect out‐of‐distribution areas that could correspond to the EZ.^16^, ^27^, ^28^ Other approaches are based on image synthesis: to predict enhanced [^18^F]FDG PET image to facilitate visual analysis^29^ or to predict the FDG normal distribution from a T1w image of the patient and compare it to the actual clinical PET. Proofs of concept of clinical relevance of last approach have been published in dementia^30^ and epilepsy.^25^ By analogy to the “SISCOM” technique in SPECT, where interictal SPECT is subtracted from ictal SPECT,^31^ DL enables a similar approach for PET: a PET, generated with DL and predicting the normal distribution of FDG, can be subtracted from the actual clinical interictal PET to reveal focal hypometabolism. We call this technique SIPCOM (Subtraction Interictal PET Co‐registered to MRI).

Here, we assessed the performance of SIPCOM to identify a matching area with the resection zone (RZ) as a surrogate marker of the EZ on preoperative [^18^F]FDG‐PET in patients with complex drug‐refractory epilepsy with a complete presurgical work‐up and subsequent epilepsy surgery. SIPCOM performance was compared to visual assessment and SPM analysis as reference methods.

## 2 MATERIALS AND METHODS

### 2.1 Patients and controls

Data were collected as part of a larger study (https://clinicaltrials.gov/ct2/show/NCT01735032) recruiting between 2012 and 2018 for which all participants provided written informed consent, and the procedures performed were in accordance with ethical standards (Lyon Sud EST IV Research Ethics Committee, approval number: N2012‐A00516‐37, 05/24/2012, NCT01735032).

Inclusion criteria for the current study were: (1) Operated adult patients who had had preoperative work‐up of DRE^2^ in our institution, (2) MRI performed at our institution and (3) [^18^F]FDG PET available, acquired on a time of flight camera and reconstructed using a 3D iterative ordered subset expectation maximization (OSEM) algorithm.

We included 20 patients (10 women, 50%). The mean age was 32 ± 7.8 years, range 17‐50. [^18^F]FDG‐PET was performed in our institution (n = 16) or another institution (n = 4). The presurgical evaluation included detailed clinical history and neurological examination, neuropsychological testing, video‐EEG recordings, clinical 3T MRI with an additional 1.5T MRI for the study protocol, and stereo‐electroencephalography (SEEG) for confirmation of the localisation of the epileptogenic zone. Additionally, we collected postoperative imaging (brain MRI or CT scans).

One SEEG expert (JJ) reviewed all the SEEG‐recorded spontaneous seizures visually to define the Seizure Onset Zone (SOZ). It was defined as the cortical area(s) exhibiting clear SEEG changes within the first 10 s of the seizure onset. SEEG changes were considered part of the SOZ when they occurred before the clinical onset of the seizure, and when they consisted of a fast‐synchronizing discharge (low voltage fast activity, or fast discharge of spikes.^32^, ^33^ Standard clinical visual analysis of PET (including coregistration with MRI) was used for planning SEEG electrode targets when a clear hypometabolic zone was detected. SIPCOM or SPM were not used in the clinical routine during the presurgical evaluation.

The surgical resection cavity was used to localize the RZ using 18 bilateral brain cortical labels: “ventral medial prefrontal” (medial orbitofrontal cortex + inferior anterior cingulate gyrus), “medial dorsal prefrontal” (medial superior frontal gyrus + superior anterior cingulate gyrus), “lateral ventral prefrontal” (inferior frontal gyrus), “lateral dorsal prefrontal” (lateral superior frontal gyrus + middle frontal gyrus), medial premotor, lateral premotor, medial central, lateral central, anterior medial temporal, anterior lateral temporal, posterior medial temporal, posterior lateral temporal, medial parietal, lateral parietal, medial occipital, lateral occipital, operculo‐insular, and temporo‐parieto‐occipital junction. The seizure‐outcome assessment was based on Engel's classification,^34^ and the outcome was categorized into good (Engel I‐II) or poor (Engel III‐IV). Pathology reports were collected when available.

The control database was composed of 37 normal adult subjects described in Ref. 35.

### 2.2 MRI and PET acquisition and reconstruction

For all participants, a T1‐weighted (T1w) MRI image was obtained on a Sonata 1.5 Tesla scanner (Siemens) with a voxel size of 1.2 × 1.2 × 1.2 mm^3^.

PET data were acquired according to recommendations.^3^ At our institution, a 122 ± 21 MBq dose of [^18^F]FDG was injected for controls and 148 ± 2.5 MBq for patients (unavailable for four patients). Data were acquired on a Biograph mCT64 PET/CT (Siemens) and reconstructed with a 3D iterative OSEM algorithm with CT‐based attenuation correction (AC) in a matrix of 200 × 200 × 109 voxels with a voxel size of 2.04 × 2.04 × 2.03 mm^3^. At the second institution, a dose of 142 ± 12 MBq of [^18^F]FDG was injected (unavailable for two patients). Data were acquired on a Discovery ST PET/CT (General‐Electrics©) and reconstructed with a 3D iterative OSEM algorithm with CT‐based AC in a matrix of 128 × 128 × 47 voxels with a voxel size of 2.34 × 2.34 × 3.27 mm^3^ except for one patient with voxel sizes of 4.69 × 4.69 × 3.27 mm^3^.

### 2.3 SIPCOM assessment

#### 2.3.1 Image preprocessing

T1w images were bias‐corrected using FSL FAST.^36^ T1w MRI and PET images were rigidly co‐registered in native space and then warped to an asymmetrical version of the Montreal Neurological Institute (MNI) template based on 152 subjects with a 1 × 1 × 1 mm voxel size via the MRI and resampled to the same voxel size using FSL.^37^ PET images were additionally smoothed by a 2 mm Gaussian filter. All values were scaled between 0 and 1.

#### 2.3.2 Image‐to‐image conditional generative adversarial (GAN) network: overview

The model was derived from the pix2pix model,^38^ which was shown to be a more efficient architecture to learn the mapping between T1w MRI and [^18^F]FDG PET than U‐net.^25^ The generator was based on 3D U‐net,^39^ with the layers of the encoding and decoding stages defined using residual units.^40^ Data in the encoder path were down‐sampled using convolutions and in the decoder path up‐sampled using transpose convolutions. The encoder consisted of five convolution layers with 32, 64, 128, 256, and 512 channels. The decoder consisted of five convolution layers with 512, 256, 128, 64, and 32 channels. Data in the discriminator was downsampled using convolution layers with 64, 128, 256, 512, and 1 channels, respectively. Parametric rectified linear units (PReLU) and batch normalization were used.

#### 2.3.3 GAN implementation details: training and performance evaluation using controls

The network was implemented using Pytorch 1.10.0 (https://pytorch.org/), Monai 0.8.0 (https://monai.io/, https://arxiv.org/abs/2211.02701) on an NVIDIA V100 GPU with 32 GB of RAM. The loss function of the generator was the average of the mean squared error multiplied by a constant lambda equal to 200 and adversarial loss. The loss function of the generator was the adversarial loss divided by 2. The optimizer was Adam. The learning rate was set to 0.0002 and kept constant.^38^ The model was trained for 1000 epochs with a batch size of 20. The network was trained using ~90% of the control data (34/37) with 32 × 32 × 32 voxel patches, testing on the remaining 3/37 controls, and the training was repeated five times with different test sets. To evaluate the predicted PET with reference to the ground truth PET image in terms of mean absolute error (MAE), peak signal‐to‐noise ratio (PSNR),^41^ and structural similarity index measure (SSIM),^41^ the predicted PET was masked using a brain mask generated using FSL BET.^42^

#### 2.3.4 Creation of the map of hypometabolic foci

Patients' normal PET was predicted using the GAN previously trained and multiplied with the ratio between the mean intensity from the clinical PET and from the predicted PET. The predicted normal PET image was then subtracted from the clinical PET. Next, the voxel values of the difference image were converted to *Z*‐scores (*Z*_*i*_ = [*X*_*i*_ − *μ*]/*σ*, where *X*_*i*_ is the voxel intensity, *μ* the mean, and *σ* the standard deviation within the brain mask). Additionally, we performed SIPCOM for each control from the test set (due to cross‐validation; n = 15) to assess the number of FP. As in previous work,^25^ the *Z* map was masked using the previously generated brain mask eroded by three voxels to reduce artifact at the low‐intensity border, and the threshold was set at *Z* > 2.33 for a cluster size of at least 1000 voxels (1 mL). The *Z*‐score value of each cluster was measured. Lastly, for SIPCOM‐positive patients, we visually assessed (1) for MRI‐positive patients, the overlap between the cluster and the lesion and (2) the overlap between the cluster and the resection cavity on postoperative imaging (MRI or if lacking CT‐scan).

### 2.4 SPM assessment

#### 2.4.1 Image preprocessing

Patient and control data were normalized using SPM12^17^ (Wellcome Department of Cognitive Neurology, Institute of Neurology) running in MATLAB 2017a (MathWorks Inc.) as previously published^35^ to the same voxel size of 2 × 2 × 2mm^3^ as in Ref. 25. Images were smoothed using an 8 mm^3^ FWHM isotropic Gaussian kernel as a final preprocessing step.^43^

#### 2.4.2 Voxel‐by‐voxel analysis

Voxel‐wise comparisons were computed between brain PET images of each patient and all control images correcting for global values via an ANCOVA per group (ie, each patient's global mean was adjusted to the controls' mean global value). Additionally, we performed SPM for each control from the test set (due to cross‐validation; n = 15) to assess the number of FP. The contrast analyzed was: patient < controls (hypometabolism). We considered clusters formed by voxels exceeding the *P* < 0.001 threshold as significant when their cluster *P*‐value was <0.05, with no minimum extent threshold.

### 2.5 Visual assessment

PET images coregistered with MRI were independently analyzed by two trained experts (AF, KOC), blinded to clinical information. Areas of hypometabolism were localized according to the previously described 18 brain cortical regions.

### 2.6 Statistical analysis

We defined a true hypometabolic cluster as a cluster whose localisation was concordant with the RZ. Discrete variables are reported as percentages, while quantitative variables are reported as mean values ± standard deviation (SD). A Cohen's kappa test was performed to assess the concordance in localisation of the RZ through PET visual analysis between expert readers. Secondly, we evaluated the performance of the PET assessment in relation to the localisation of the epilepsy (temporal vs extra‐temporal epilepsy), the MRI findings (MRI‐positive vs MRI‐negative), the postsurgical seizure outcome (good vs poor) and according to the PET scanner (Siemens vs GE). We additionally computed the recall and the precision for both methods. Pairwise comparisons between methods were performed using Fisher's exact tests The two‐tailed significance level was set at *P* = 0.05.

## 3 RESULTS

### 3.1 Population description

Clinical characteristics are displayed in Table 1. MRI findings were considered negative (cryptogenic epilepsy) in eight cases (40%), whereas a lesion was identified on MRI (symptomatic epilepsy) in the 12 others (60%). Eight patients (40%) had a left‐sided EZ, and 14 patients (70%) had temporal lobe epilepsy. After surgery, the mean follow‐up was 20.6 ± 14.8 months (range, 2‐48); 14 patients (60%) had a good outcome (Engel class I or II).

**TABLE 1:** Patients' characteristics.

Number	Age	Gender	MRI	Lobar localization	Anatomopathological findings	Follow‐up (months)	Engel class
1	17	M	R central FLAIR hyperintensity	R Central	FCD 2A	48	1a
2	41	F	R posterior temporal cavernoma + R hippocampal sclerosis	R Temporal	FCD 3A	28	1a
3	41	M	Negative	R Temporal	FCD 3A	4	1a
4	38	M	Bilateral hippocampal sclerosis	R Temporal	Nonspecific gliosis	10	1a
5	33	F	L temporal pole atrophy + L amygdala FLAIR hypersignal	L Temporal	Nonspecific gliosis	2	1a
6	29	M	L temporal pole blurring + L temporal pole atrophy	L Temporal	Nonspecific gliosis	24	1a
7	33	M	Negative	L Temporal	Nonspecific gliosis	32	1a
8	25	M	Negative	R TPO	Normal	35	1a
9	24	F	R parieto‐occipital scar + R hippocampal sclerosis	R Temporal	NA	13	1a
10	36	F	Negative	R Temporal	NA	3	1a
11	30	F	Left parietal blurring + L parietal FLAIR signal hyperintensity + L transmantle sign	L Parieto‐occipital	NA	4	1a
12	31	F	Negative	R Temporal	FCD 3A	40	1b
13	21	F	L insular FLAIR hypersignal	L Temporal	Normal	12	2b
14	40	F	L hippocampal sclerosis	L Temporal	FCD 1C	38	2b
*15*	*50*	*M*	*L temporal pole FLAIR hypersignal + L Temporal pole atrophy*	*L Temporal*	*FCD 3D*	*7*	*3a*
*16*	*30*	*M*	*Negative*	*R Frontal*	*Normal*	*31*	*3a*
*17*	*26*	*M*	*R hemispheric atrophy + R amygdala, hippocampus FLAIR hypersignal*	*R Temporal + TPO*	*Nonspecific gliosis*	*17*	*3a*
*18*	*26*	*F*	*Negative*	*R Frontal*	*Nonspecific gliosis*	*36*	*3a*
*19*	*28*	*M*	*Negative*	*R Central*	*Nonspecific gliosis*	*3*	*4b*
*20*	*34*	*M*	*L temporal pole atrophy*	*L Temporal*	*Nonspecific gliosis*	*25*	*4b*

*Note*: Lateralisation/localisation: at the lobar level; more granular localisation per patient is provided in Table 2. Italics: patient's outcome was Engel 3 or 4.

Abbreviations: F, female; FCD, focal cortical dysplasia; L, left; M, male; NA, non available; R, right; TPO, temporo‐parieto‐occipital.

### 3.2 Evaluation of the predicted PET image

**FIGURE 1:**
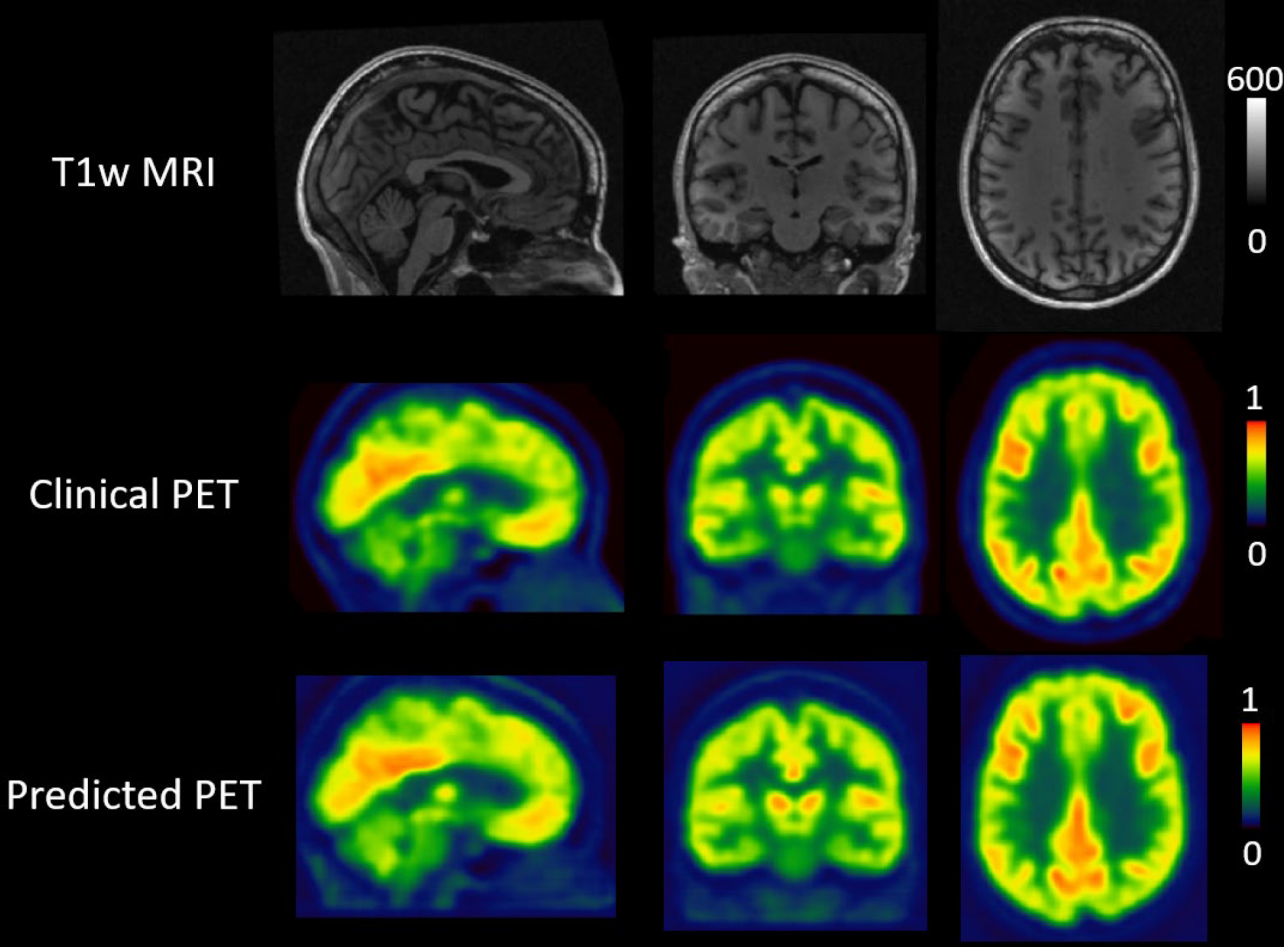
GAN results for one representative control subject. The first row shows the T1w MRI, the second row the clinical [^18^F]FDG PET, and the third row the predicted [^18^F]FDG PET, ie, the output from the generative adversarial network. From left to right, sagittal, coronal, and transverse slices are shown for each set.

The conditional GAN was successfully trained to learn the mapping from the T1w to the [^18^F]FDG PET. Across controls, the mean MAE was 0.0042 ± 0.0021, the mean PSNR was 35.14 ± 3.846, and the mean SSIM was 0.991 ± 0.0025. Figure 1 showcases the result for one representative control subject (MAE 0.0044, PSNR 34.85, SSIM 0.992).

### 3.3 Semi‐quantitative analysis: SPM and SIPCOM

SPM and SIPCOM results are summarized in Table 2. An area concordant with the RZ was detected in 12/20 patients (60%) using SIPCOM vs 7/20 patients (35%) using SPM (*P* = 0.015). All those detected using SPM were also detected using SIPCOM. The recall for SPM vs SIPCOM is 0.35 vs 0.48 and the precision is 0.13 vs 0.21. For the 12 SIPCOM concordant patients, there was an overlap between the cluster defined using SIPCOM and the resection cavity on postoperative imaging.

**TABLE 2:** Results for each subject according to each method.

#	SEEG	Surgery	MRI	PET visual analysis	PET SPM	PET SIPCOM
1	MC + LC + MP + LPr R	*Idem*	MP + LPr + MC + LC R	MP + LPr + MC + LC R	LPr + LC R	LPr + LC + MP + MC R
2	AML + ATL R	*Idem*	PLT R	AML + ATL R	N	N
3	AML + ATL R	*Idem*	N	N	N	N
4	AML + ATL R	*Idem*	AML R + AML L	AML + ATL R	AML + ATL R	AML + ATL R
5	AML + ATL + OI L	*Idem*	AML + ATL L	AML + ATL L	ATL L	AML + ATL L
6	AML + ATL L	*Idem*	ATL L	AML + ATL L	N	AML + ATL L
7	AML L + ATL L + ATL R	**AML + ATL L**	N	AML L	N	N
8	PLT + LP + TPO R	*Idem*	N	AML + ATL + PLT R	N	PLT R
9	AML + ATL R	*Idem*	AML + PLT R	AML + ATL + PLT R	AML + ATL + PLT R	AML + ATL + PLT R
10	AML + ATL + PMT + PLT R	*Idem*	N	N	ATL + PLT R	ATL + PLT R
11	LP + LO L	*Idem*	LP + LO L	LP + LO L	N	LP + LO L
12	ATL R	*Idem*	N	ATL R	N	ATL R
13	*PLT + ATL + OI L*	*Idem*	*OI L*	*AML + ATL + PLT + OI L*	*N*	*N*
14	*AML + ATL L*	*Idem*	*AML L*	*AML + ATL L*	*N*	*N*
*15*	*AML + ATL L*	*Idem*	*ATL L*	*AML + ATL L*	*ATL L*	*ATL L*
*16*	*VLPF + DLPF + LPr + MP R*	***MP + LPr R***	*N*	*N*	*N*	*N*
*17*	*ATL + PLT + TPO R*	*Idem*	*ATL + PLT R*	*ATL + PLT + OI R*	*ATL + PLT + OI R*	*ATL + PLT + OI R*
*18*	*VLPF + OI R*	*Idem*	*N*	*VLPF + OI R*	*N*	*VLPFR*
*19*	*MC + LC R*	*Idem*	*N*	*MC + LC R*	*N*	*N*
*20*	*AML + ATL L*	*Idem*	*AML + ATL L*	*AML + ATL L*	*N*	*N*

*Note*: Italic: outcome Engel 3,4. Bold: surgery different from SEEG.

Abbreviations: AML, Anterior medial temporal; ATL, anterior lateral temporal; DLPF, dorsal lateral prefrontal; LC, lateral central; LO, lateral occipital; LP, lateral parietal; LPr, lateral premotor; MC, medial central; MP, medial premotor; OI, operculo‐insular; PLT, posterior lateral temporal; PMT, posterior medial temporal; SEEG, stereotaxic electroencephalogram; TPO, temporo‐parieto‐occipital junction; VLPF, ventrolateral prefrontal.

Results were similar in the subgroup of good outcome patients (Engel I‐II) as the RZ site was detected in 9/14 patients (64%) using SIPCOM vs 5/14 patients (36%) using SPM. For other Engel outcomes (III, IV), SIPCOM localized the RZ in 3/6 patients (50%) vs 2/6 patients (33%) for SPM.

**FIGURE 2:**
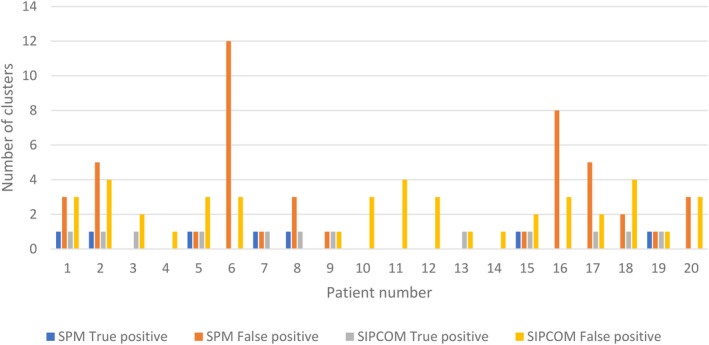
Histogram showing the number of true and false positive clusters for each patient using statistical parametric mapping or Subtraction Interictal PET Co‐registered to MRI.

In the whole group, the average number of false positive (FP) clusters was 2.2 ± 1.3 using SIPCOM and 2.3 ± 3.1 using SPM (*P* > 0.5), ranging from 0 to 4 in SIPCOM and 0‐12 in SPM (Figure 2). Among all the FP clusters, 63% were localized in four bilateral cortical brain regions (lateral dorsal prefrontal 25%, ventral medial prefrontal 16%, medial parietal 11% and medial occipital 11%). All the other regions yielded FP rate below 5% and five bilateral regions did not include any FP (medial dorsal prefrontal, medial premotor, lateral premotor, medial central and posterior lateral temporal). Only 4 FP clusters (9%) were included in the propagation zone: subject #1 a left lateral dorsal prefrontal cluster, subject #16 a right ventral medial prefrontal cluster, subject #17 a right operculo‐insular cluster and subject #20 a right anterior lateral temporal cluster. For comparison, in the control group, the average number of FP clusters in controls was 1.9 ± 1.4 using SIPCOM and 2.9 ± 3.2 using SPM, ranging from 0 to 6 in SIPCOM and 0‐12 in SPM.

The mean *Z*‐score in the resection zone was 4.8 ± 0.98 vs 4.5 ± 2.46 in the false‐positives clusters (*P* = 0.64). In 41% of the cases, the value of the *Z* score in the resection zone was higher than the value of the false positives.

**FIGURE 3:**
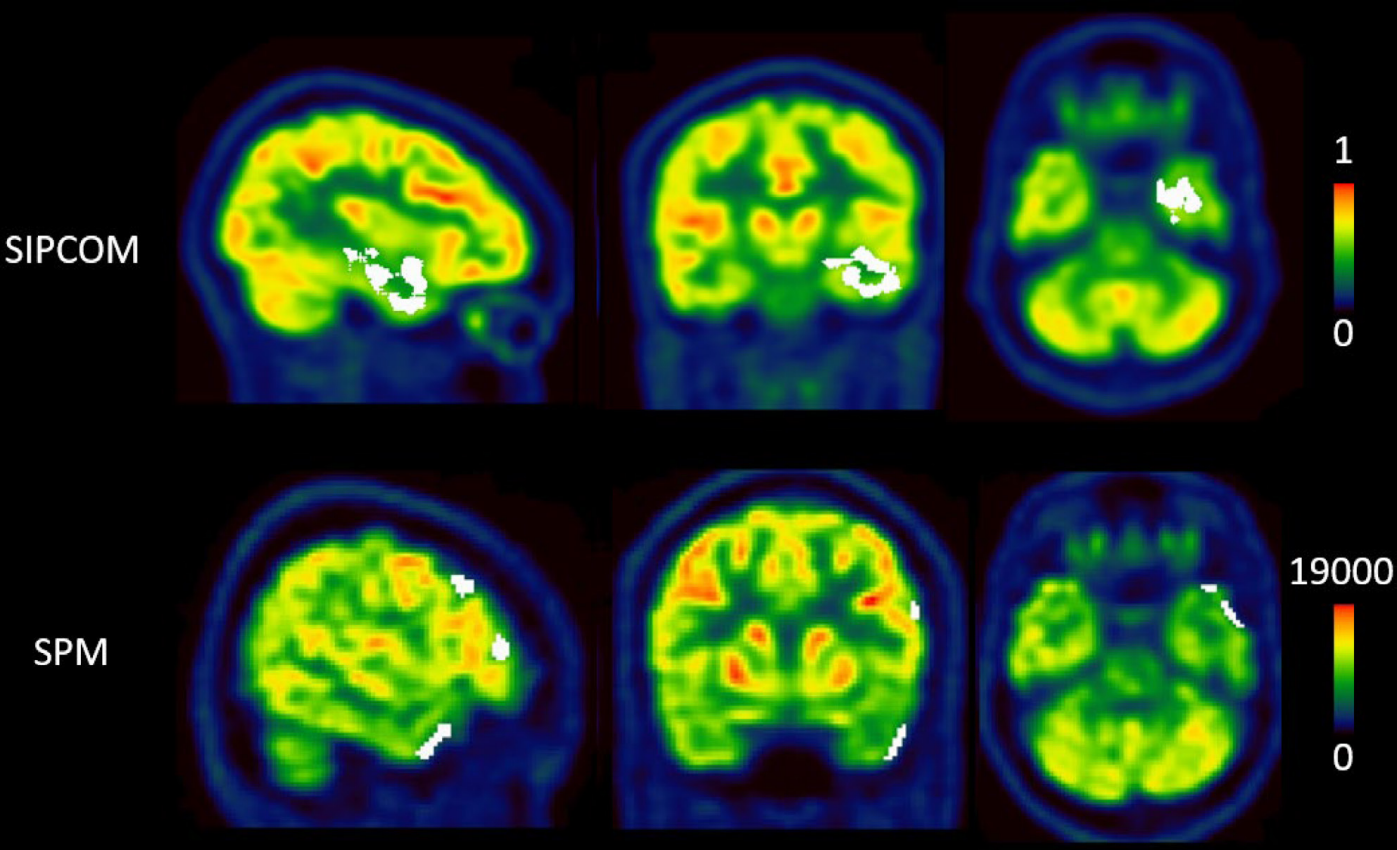
Result of a concordant case between statistical parametric mapping (SPM) and Subtraction Interictal PET Co‐registered to MRI (SIPCOM) analysis (patient #5). The first row displays the [^18^F]FDG PET superimposed with an abnormal cluster (in white) defined using SIPCOM. The second row displays [^18^F]FDG PET superimposed with abnormal clusters defined using SPM. Cluster size and localisation did not overlap between analyses. The frontal clusters seen with SPM (false positives) likely reflect the patient's individual anatomy and are therefore not seen with SIPCOM.

**FIGURE 4:**
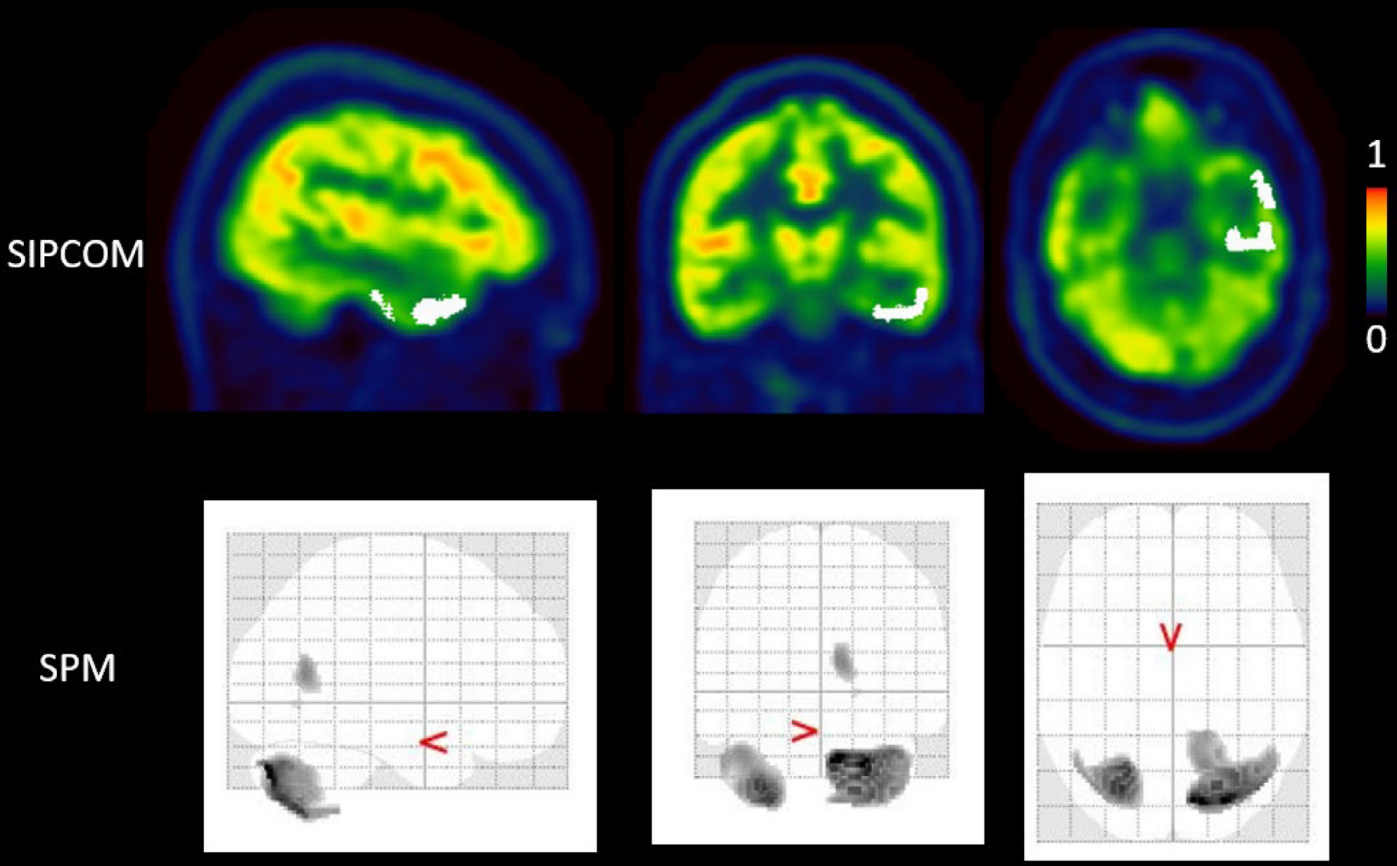
A case (patient #6) with discordant results for Subtraction Interictal PET Co‐registered to MRI (SIPCOM) and statistical parametric mapping (SPM). The first row displays the [^18^F]FDG PET superimposed with an abnormal left temporal cluster defined using SIPCOM. The second row displays the maximum intensity projection result of the SPM analysis for the same patient, showing no cluster of hypometabolism in the left temporal lobe.

One example each of concordant and discordant results between SPM and SISCOM for two different left TLE cases is shown in Figures 3 and 4. Figure 3 highlights that whereas both methods correctly localized the RZ, SIPCOM identified a more extensive cluster than SPM. For the patient shown in Figure 4, SIPCOM correctly identified the temporal cluster but not SPM. Overall, in TLE, SIPCOM correctly localized the RZ in 9/14 patients (64%) vs 6/14 patients (43%) using SPM. In ETLE, SIPCOM correctly localized the RZ in 3/6 patients (50%) vs 1/6 patients (17%) using SPM. In MRI‐positive patients, the SIPCOM concordance rate was 8/12 (67%) vs 6/12 (50%) using SPM, and in MRI‐negative patients, SIPCOM positivity was 4/8 (50%) vs 1/8 (13%) in SPM. Among the 8 SIPCOM‐positive patients with lesioned MRI, the SIPCOM cluster overlapped with the lesioned area in 7 (88%). Only for subject #4 with bilateral hippocampal sclerosis, the clusters were mainly located peripheral to the lesions (larger on the right): in the temporal pole and the inferior gyrus of the temporal lobe.

Of the three [^18^F]FDG PETs normal on visual reads, one (patient #10) with TLE was positive (33%) using both SPM and SIPCOM.

In the subgroup of PET acquired using Siemens PET/CT, SIPCOM localized the RZ in 9/16 patients (60%) vs 5/16 patients (38%) in SPM, whereas for GE PET/CT images, SIPCOM localized the RZ in 3/4 patients (75%) vs 2/4 patients (50%) in SPM.

### 3.4 Visual analysis

Visual PET analysis results are summarized in Table 2. Hypometabolic areas concordant with the RZ site were detected in 17 patients (85%) (*P* = 0.54 vs SIPCOM). PET visual analysis was negative in three patients (15%). Inter‐reader agreement for localisation of the anomaly was excellent with Cohen's *k* = 1. In MRI‐positive patients, visual analysis was positive in 12/12 patients (100%), while in MRI‐negative patients, it was positive in 5/8 patients (63%).

## 4 DISCUSSION

We evaluated the performance of the SIPCOM method to detect the RZ in a cohort of complex DRE patients. The SIPCOM concordance rate was higher, at 60%, than SPM, at 35%, but lower than visual analysis, at 85%. It should be noted that we optimized visual analysis by systematically coregistering PET onto MRI, which is known to improve performance.^7^, ^8^, ^10^ Visual analysis results could be further improved using a simultaneous PET‐MRI scanner.^44^

The computer‐aided methods did detect the RZ in one of the three patients in whom both MRI and PET had been unremarkable, a result that is potentially very clinically relevant.

SIPCOM results were relatively similar in different sub‐groups with concordance rates of 64% in TLE vs 50% in ETLE, 67% in MRI‐positive patients vs 50% in MRI‐negative patients, 56% with Siemens PET/CT vs 75% (3/4) with General Electric PET/CT.

In our study, SPM concordance rates were towards the lower end of previously published results which ranged from 20%^26^ to 86%.^6^ Overall, there is no clear pattern to explain this large range of previously published SPM results which are difficult to compare due to various differences: in types of cohorts patients selected by epilepsy subtypes (TLE^16^ or ETLE^15^) or without selection,^5^, ^24^, ^26^ adults^22^ or children,^6^, ^16^, ^20^ with different gold standards (ie, good outcome after surgery,^5^, ^6^, ^15^, ^20^, ^26^ SEEG,^19^, ^23^ or noninvasive work up^16^, ^22^), using different SPM versions (SPM99,^15^ SPM5,^19^ SPM8,^16^, ^22^ SPM12^26^), different SPM statistical parameters (*P*‐value, cluster size threshold),^20^, ^21^ different methods to select the cluster (largest volume,^26^ highest *T*‐value,^6^, ^15^ the *P*‐value at the cluster level^16^), different definitions of the EZ (results of the presurgical work‐up,^23^ the SEEG,^19^ or the resected area^24^), and finally the control databases, ranging in size from 13^20^ to 55^25^ and composed from control subjects^21^, ^25^ or patients undergoing a whole body scan for an unrelated indication.^23^

The lower performance of both SPM and SIPCOM over visual analysis in our study may be partly explained by the preprocessing steps (smoothing and anatomical registration of the image for SPM). Those steps could hamper the lesion detectability in opposition to visual analysis which is mainly driven by asymmetrical analysis.

SIPCOM concordance rate was higher than SPM (25%). One possible explanation is the reference used for the statistical comparison. In voxel‐wise statistics such as SPM, data preprocessing requires a morphological transformation of PET images to a standardized template, followed by substantial spatial smoothing which is performed to increase the signal‐to‐noise ratio^18^ and compensate for anatomical variation between the subject and the group of controls. Comparison is then against an identically processed normal control database which will have a certain normal distribution which will vary by region.^35^ In contrast, the SIPCOM statistics are based on the voxel value compared to the intra‐subject variability across the brain of the patients themselves, ie, mean and standard deviation of the difference image, contained in the *Z*‐score. In SIPCOM, the reference to a normal database is implicit, contained in the GAN training from a control database and the patient's native space is used, reducing the need for smoothing. Hence SIPCOM could be seen as more personalized. While interpretation of the results of semi‐automatic methods still depends on users' understanding of the methodology and potential artifacts, those methods stand to reduce subjective bias in interpretation, in order not to overlook any area of the brain in the PET image. Additionally, such methods can offer a homogeneous reference level of PET assessment between readers or during their training.

Compared to a previous study in epilepsy using predicted PET synthesized from MRI as a reference for subtraction,^25^ here the SIPCOM FP rate was lower, similar to that of SPM. At least two methodological points may explain this difference: (1) the predicted PETs generated in our work had better image quality metric values than in the previous study; they are also the best compared to previous studies,^25^, ^30^, ^45^, ^46^ and (2), in our work, the predicted PET global mean voxel values were normalized to fit the global mean of the actual PET of the subject, an innovation inspired by the SPM scaling process. Taken together, these two improvements resulted in more accurately predicted PETs which may explain the smaller number of FP findings. However, FP were still present and only 9% could be related to seizure propagation pathways.^47^ Lastly, the epilepsy cohorts were different (operated patients here vs patients coming for PET, ie, much earlier in the presurgical workup) as well as the reference standards (RZ in this work vs a visually abnormal scan in previous work).

Our work has several limitations. Firstly, it is a retrospective study based on small sample size. A large prospective study should confirm these initial results and evaluate in which types of focal epilepsy the method is the most beneficial (eg, lesional vs nonlesional, suspected malformations of cortical development). Secondly, only 60% of the patients achieved a good outcome (Engel class 1 or 2); but the results were similar in the subgroup of good outcome patients vs the whole cohort as the RZ site was detected respectively in 64% vs 60% using SIPCOM and 35% vs 36% using SPM. Interestingly, even when SIPCOM and SPM are both able to localize the RZ according to the anatomical grouping used here, the cluster shape is not identical (Figure 2); shape differences and their impact could be investigated. Thirdly, both computer‐assisted methods are highly dependent on choices of parameters used in the preprocessing steps and analyses as well as image characteristics. For example, differences in the MRI field, MRI manufacturers and parameters used to acquire the T1w images can alter the GAN performance to predict the PET image and limit the portability of the method. The impact of different T1w images needs to be carefully evaluated as it may imply tuning the GAN, using transfer learning so that new input MRI images can be used. Lastly, another work perspective would be to port SIPCOM for use with children but as for SPM, a dedicated normal database would be necessary.

## 5 CONCLUSION

In a group of DRE patients who underwent presurgical work‐up including SEEG and were operated on, SIPCOM was able to localize the RZ in 60% of the patients vs 35% using SPM. Both detected the RZ in one of three patients with no abnormality on visual analysis, suggesting that they may be a complementary procedure. SIPCOM's impact in particular should now be validated in larger prospective studies.

## AUTHOR CONTRIBUTIONS

JJ participated in clinical data collection and analysis. SR, MG participated in clinical data collection. KOC, SB performed the clinical data collection and PET visual analysis. AF conceived the study, performed the visual, the SIPCOM and SPM analysis. AH participated in data analysis (SPM). SNY helped to perform the SIPCOM implementation. All authors contributed to writing, read, and approved the final manuscript.

## CONFLICT OF INTEREST STATEMENT

None of the authors has any conflict of interest to declare. We confirm that we have read the Journal's position on issues involved in ethical publication and affirm that this report is consistent with those guidelines.

## ETHICS STATEMENT

“We confirm that we have read the Journal's position on issues involved in ethical publication and affirm that this report is consistent with those guidelines.” All procedures performed in the study and involving human participants were performed in accordance with the ethical standards of the institutional and/or national research committee and with the principles of the 1964 Declaration of Helsinki and its later amendments or comparable ethical standards. All participants provided written informed consent, and the procedures performed were in accordance with ethical standards (Lyon Sud EST IV Research Ethics Committee, approval number: N2012‐A00516‐37, 05/24/2012, NCT01735032).

## Data Availability

PET data from controls can be requested for bona fide research (merida@cermep.fr; see Mérida I et al. *EJNMMI Res* 2021). The PET data from patients can be requested through a joint institutional ethics agreement.
